# Wives! To the Rescue!!

**Published:** 1874-01

**Authors:** 


### WIVES! TO THE RESCUE!!

THERE is a man in Wall street, who could have raised half a million of dollars on his own notes any day, and so recently as a month ago; yesterday he mortgaged his magnificent mansion for thirty thousand dollars, because he could not raise that sum.

A bank president showed a letter the other day containing fifty dollars from a friend, who had borrowed five hundred, to be returned whenever demanded.

There is many a man in New York this winter who could have handed his wife a hundred dollars for the weekly household expenses, a year ago, easier than he can give her ten, or even five, to-day. There is many a wife who could save her husband from ruinous sacrifices or failures if she would for a short time do with one fifth the money that she had hitherto; and she could do it if she would.

There are wives and daughters in New York to-day who have been accustomed to their monthly allowance, who would feel as much outraged not to receive it, to the last farthing, as would a workman in our down town manufactories. Some husbands, knowing this, would rather let a note pass unpaid than to face the storm which they know would arise if they failed of promptitude in their own family; they know that they would be met with the unreasoning rejoinder, "You promised it, and if you knew you couldn't comply with it you should not have made the promise." This is the logic of many a boarding school wife, and perhaps of those who never had any schooling worth the name, and yet think themselves intelligent.

There are women who say they could not help their husbands' failures; they were not down town to look after matters, and knew nothing about business affairs. There is a falsity here. In any business community there are men who, for the want of fifty dollars, had to go to protest, and to the wall, and then to despair, and the bottle, and the gutter. In cases not a few the wife could have prevented all this by cutting down expenses promptly and sharply.

How "promptly and sharply?" Pay every servant off and dismiss them on the instant. The food of a servant costs three dollars a week in New York, besides the wages. Dismiss your butcher, and grocer, and baker, and iceman. Spend not a cent of money for anything but milk, "grits," or Carr Graham flour; a dollars' worth of these will abundantly feed a family of five for one day, and at the end of a month hence they will be in better health, and weigh more than at the beginning. Any woman who has force of character—who has the true grit of a wife—would not only adopt such a course promptly, but would do it with a grand, brave heart, and feel a pleasure and a moral elevation in thus coming to the rescue of her husband, and is worth more than money. There are a thousand families in New York to-day who could reduce their daily household outlays from ten dollars to one, and when the "panic" is over would feel proud of what they had done, and it would be a pardonable boast to them for the remainder of life.

If all the families in New York who *could* do without another stitch of new clothing for the next six months would only do so, there would be a million of money saved to pay off their husband's debts; and the million would go round in liquidating other debts, to the amount of fifty millions of dollars, before the spring time comes, and the beautiful May and the rosy leaf of June.

